# Chronic obstructive pulmonary diseasés impact on the affected person and next of kin: A mixed methods study

**DOI:** 10.1177/17423953231175971

**Published:** 2023-05-16

**Authors:** Helena Johansson, Katarina Berg, Lise-Lotte Jonasson, Carina Berterö

**Affiliations:** 1 Department of Medical Specialist in Motala, Department of Health, Medicine and Caring Sciences, Linköping University, Linköping Sweden; 2 571308 Department of Health, Medicine and Caring Sciences, Linköping University, Linköping Sweden; 3 Department of Nursing, School of Health and Welfare, Jönköping University, Jönköping Sweden

**Keywords:** caregiver burden, chronic obstructive pulmonary disease, sense of coherence, support, symptom burden

## Abstract

**Objectives:**

Severe chronic obstructive pulmonary disease affects and changes the lives of both affected persons and next of kin. There is a need for support and a sense of coherence to manage the life situation and minimize the symptom and caregiver burden. The aim of this study was to diverge or converge views of symptom burden, caregiver burden, the need for support, and sense of coherence in persons with chronic obstructive pulmonary disease and their next of kin to gain a deeper and broader knowledge and understanding.

**Methods:**

A mixed methods study with data from interviews and four validated questionnaires from persons affected by chronic obstructive pulmonary disease in GOLD stages III and IV and their next of kin.

**Results:**

Questionnaires from 112 persons affected by chronic obstructive pulmonary disease, and 71 next of kin, together with 25 and 21 interviews, show that; there is a difference between estimated symptoms and caregiver burden and experiences expressed in their own words. There is also a defect regarding meaningfulness, comprehensibility, and manageability affecting daily life. Symptoms and caregiver burden, together with the sense of coherence, strengthen the need for support.

**Discussion:**

The complexity of the life situation leads to a need for supportive interventions to strengthen internal and external resources.

## Introduction

Severe chronic obstructive pulmonary disease (COPD) in GOLD-stages III–IV is a chronic, inflammatory, and irreversible lung disease with Forced Expiratory Volume 1 s (FEV_1_) below 50%. The affected person has symptoms such as dyspnea, cough and increased mucus production, which affect the daily life^1^ with a symptom burden defined as limitations in daily activities and psychological suffering.^2^

Persons with severe COPD have a changed life situation, a need for balanced support from the next of kin if possible, and a need to live life one day at a time.^3^

Through estimations of the affected persons’ functional status, its effect on daily life and social interaction the caregiver burden can be indicated.^4^ The caregiver burden for the next of kin, can be defined as an imbalance between stressors and possibilities to cope with the situation.^5^ This imbalance may have an impact on both physical and psychological aspects of the person who takes care of a person affected by a chronic illness.^6^ Next of kin experience caregiver burden in changed family roles and activities. They must put their own life on hold, and it is emotionally stressful to stand aside and observe the breathing problems as the disease and the symptoms worsen.^7^ COPD affects both the persons with COPD and their next of kin and there is a changed life situation.^3,7^ To manage this situation there is a need for comprehensibility, manageability, and meaningfulness of the situation, all of which are dependent on and affected by internal and external resources. The perception of how understandable the situation is, gives a sense of coherence.^8^

Persons with COPD have a need for information about self-care.^9,10^ The next of kin need to be included in the self-care approach, and thereby they need support related to symptoms, treatment, and disease progression. They also need support with managing the situation and helping the affected person. Such support contributes to making the situation understandable and manageable.^7^ A way to develop this support and help the affected persons and their next of kin to manage their daily life and life situation is to plan for a person-centered supportive intervention. Before designing person-centered interventions there is a need for detailed knowledge and understanding about both the affected person's and their next of kins’ life situation both estimated and expressed in the person's own words. Therefore, the aim of this study was to diverge or converge views of symptom burden, caregiver burden, the need for support and sense of coherence in persons with COPD and their next of kin to gain a deeper and broader knowledge and understanding.

## Material and method

### Design

This study had a concurrent mixed methods design enabling simultaneous answers to confirmatory (quantitative) and exploratory (qualitative) questions in order to divergent or convergent the results from these both methodological perspectives.^11^

### Participant selection and settings

**Figure 1.**
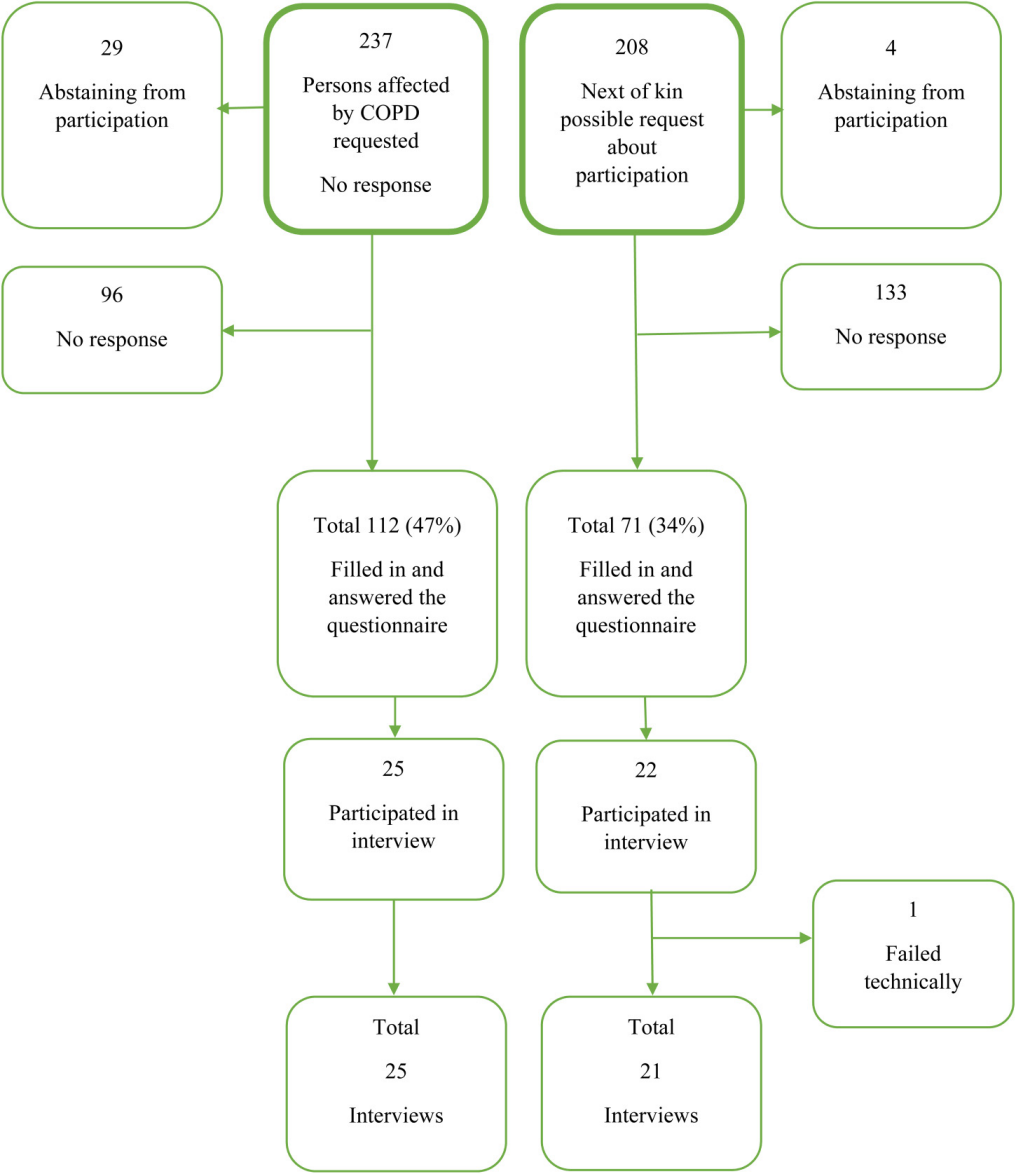
Flowchart showing the inclusion and participation of affected persons with chronic obstructive pulmonary disease (COPD) and their next of kin.

Data were collected from persons diagnosed with COPD in GOLD stages III and IV and their next of kin. Persons with COPD were recruited from the pulmonary reception at seven hospitals in the south of Sweden between March 2015 and December 2019 (Figure 1). The persons affected by COPD were verbally informed about the study by a physician or a nurse. If they were interested in participating, they were given a package containing written information about the study, an informed consent form and the questionnaires. There was also an invitation to participate in an interview study and a pre-paid envelope to send the material back. The affected person (Figure 1) also received a similar package to pass on to a person acknowledged as a next of kin selected by the person himself. The informed consent form and questionnaires were completed and returned to the first author. Inclusion criteria were being a person with COPD in stages III or IV and next of kin acknowledged by the affected person. The exclusion criterion was an ongoing exacerbation for the persons affected by COPD, while for the next of kin there were no exclusion criteria. In the recruitment process, no reminders were sent out due to which the research team did not know who was recruited and who was chosen as a next of kin. No contact information was received before the questionnaire package was returned. All data, both quantitative and qualitative, were collected in parallel.

The Ethics Review Board in Sweden approved the study; record no 2014/394-3.

The research was conducted in line with the Declaration of Helsinki.^12^

### Questionnaires

#### Symptom burden

Symptom burden was measured by Received Memorial Symptom Assessments Scale (RMSAS) as an assessment of physical and psychological symptoms and their prevalence for persons with COPD.^13^ Twenty-one symptoms are assessed from the perspective of frequency, severity, and distress on a four- and five-point numeric scale. Frequency is rated 1–4 (1 rarely, to 4 almost constantly), severity 1–4 (1 light, to 4 very severe) and distress 0–4 (0 not at all, to 4 very often). The initial step calculates a score for each symptom. If a symptom is not experienced, the score is 0. If a symptom is experienced, the score is determined as the average of the scores on frequency, severity, and distress. The reliability of RMSAS has a Cronbach´s alpha value of 0.86.^13^

#### Caregiver burden

Caregiver burden was measured by Caregiver Reaction Assessment (CRA), which measures next of kin's ability to care for a person with illness, and positive or negative effects on the next of kin's life situation.^14^ The caregiver burden is assessed with 24 statements on a five-point numeric scale 1–5 (1 strongly disagree, to 5 strongly agree) in the original version. The individual statements are assessed in five subscales: caregiver esteem, lack of family support, impact on finances, impact of schedule and impact on health. In this study a revised Swedish version was used,^15^ where the neutral item 3 = agree nor disagree, was deleted and therefore in this study, there was a four-level Likert type response; ‘Does not apply at all’, ‘Does not apply very well’, ‘Applies pretty much’, ‘Applies completely’. The scale consisted of 24 items estimated from 1 to 4 with obtainable scores ranging between 24 and 96. Before calculation, five items were reversed. Low scores indicate high levels of caregiver burden, and high scores indicate low levels of burden. Each subscale was merged to a sum score, which was divided by the number of items, which reflected the mean-item score with a range from 1.0 to 4.0. The CRA has strong internal consistency for total scale scores, alpha = 0.91^14^ and the Swedish version 0.76.^15^

#### Support

Support was measured with the Social Support Questionnaire 6 (SSQ6) considering two dimensions of social support: availability, and satisfaction. The affected persons and next of kin reported the number of existing supportive persons and assessed their satisfaction with the support on a six-point Likert scale 1–6 (1, very dissatisfied to 6, very satisfied). Every item was calculated as an item score with a mean of the number of supportive persons and a satisfaction score. The instrument had a Cronbach's alpha of 0.90–0.93.^16^

#### Sense of coherence

Sense of coherence 13 (SOC13) measures the relationship between health and illness in respect of comprehensibility, manageability and meaningfulness.^17^ Comprehensibility is the sense that one's internal and external environment is ordered: consistent, structured and clear. Manageability means that resources are required to deal with the situation and that these resources are available. Meaningfulness is experienced if the challenges feel worth getting involved in.^8^ The SOC consists of 13 questions on a seven-point Likert-type scale with responses ranging from 1 to 7 (1, very often to 7, very seldom or never). Possible scores range between 13 and 91. A low score indicates a low SOC. The instrument has a Cronbach's alpha of 0.82–0.95.^18^ The instrument also has a well-documented validity.^17–19^

### Qualitative interviews

Semi-structured interviews were conducted with 25 persons affected by COPD in GOLD stages III (*n* = 16) and IV (*n* = 9)^3^ and 21 next of kin, (11 and 10, respectively).^7^ The interviews were performed at a place and time chosen by the interviewees, were digitally recorded and transcribed verbatim. All interviews were conducted by the first author. The interviews with persons affected with COPD had a mean length of 42 min, and interviews with next of kin had a mean length of 33 min.

### Data analysis

Quantitative data was analyzed with frequencies and means^20^ for differences between affected persons in COPD stages III and IV and affected persons with next of kin. When comparing demographic variables with the group of affected persons and next of kin and differences between the groups, an independent *t*-test was used (*p* = .05). The data were computed with the SPSS software, version 28.0. Qualitative data from the transcribed interviews were analyzed inductively with the six-step reflexive thematic analysis.^21,22^ The analysis was led by the first author but was coded and analyzed by all authors separately following the steps, and discussed for consensus.^3,7^

**Figure 2.**
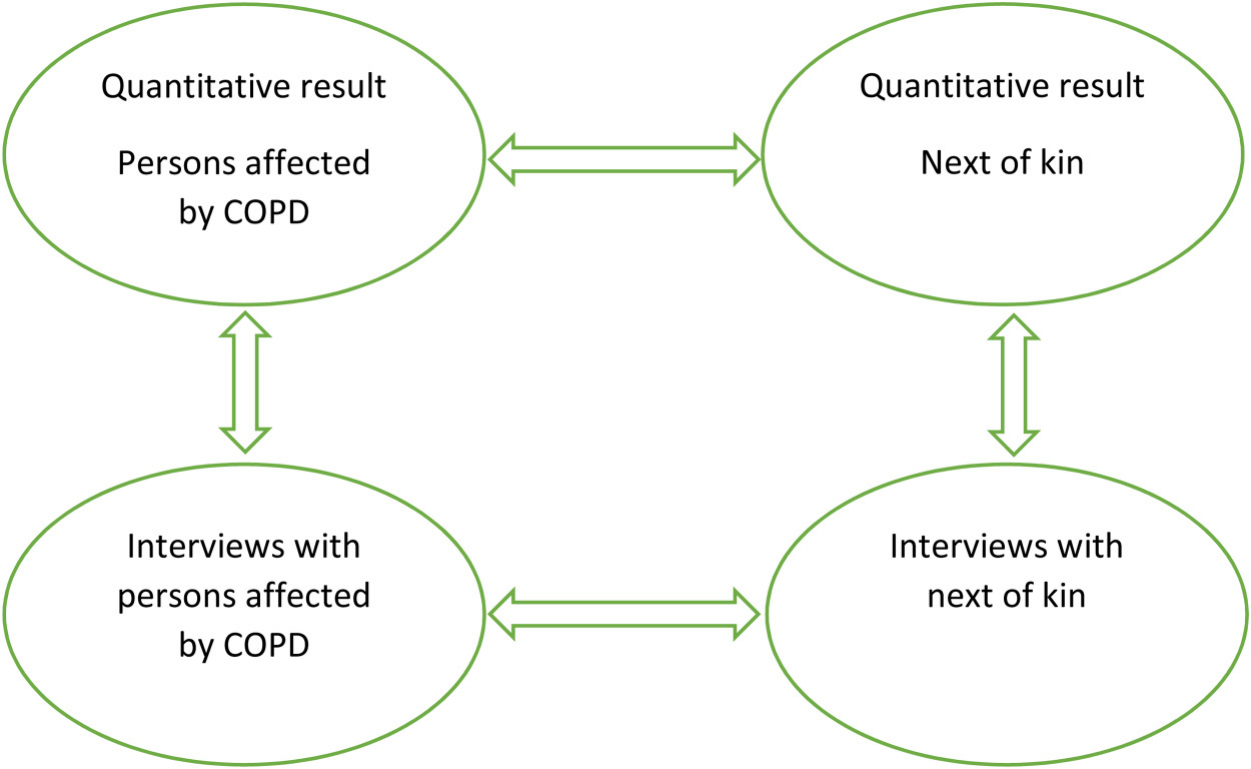
The mixed methods analysis process.

The qualitative results^3,7^ and primary quantitative results were first analyzed separately and then integrated and interpreted using mixed methods and are shown according to the aim. The mixed methods involved reallocating the results of the qualitative thematic analysis with quantitative results. In order to diverge or converge the qualitative and quantitative results they were compared and contrasted, and result from affected persons were compared and contrasted with result from next of kin. This procedure was aiming for confirmation, identifying contradictions and/or new perspectives^11,23^ (See Figure 2).

## Results

### Participants

Participants were persons diagnosed with COPD in GOLD stages III and IV and their next of kin (Table 1).

**Table 1.** Characteristics of the participants answering the questionnaires and participants in interviews.

		Questionnaires		Interviews	
Participants	Characteristics	Persons with chronic obstructive pulmonary disease (COPD) *n* = 112	Next of kin *n* = 71	Persons with COPD *n* = 25	Next of kin *n* = 21
Gender *n* (%)	Female	71 (63)	42 (59)	14 (56)	14 (67)
	Male	41 (37)	29 (41)	11 (44)	7 (33)
GOLD					
Stage affected	III	58 (52)	35 (49)	16 (64)	11(52)
Person *n* (%)	IV	54 (48)	36 (51)	9 (36)	10 (48)
Age range (m)	Year	44–87 (72)	41–81(65)	58–82 (71)	46–77 (62)

### Results from the questionnaires

#### Symptom burden

**Figure 3.**
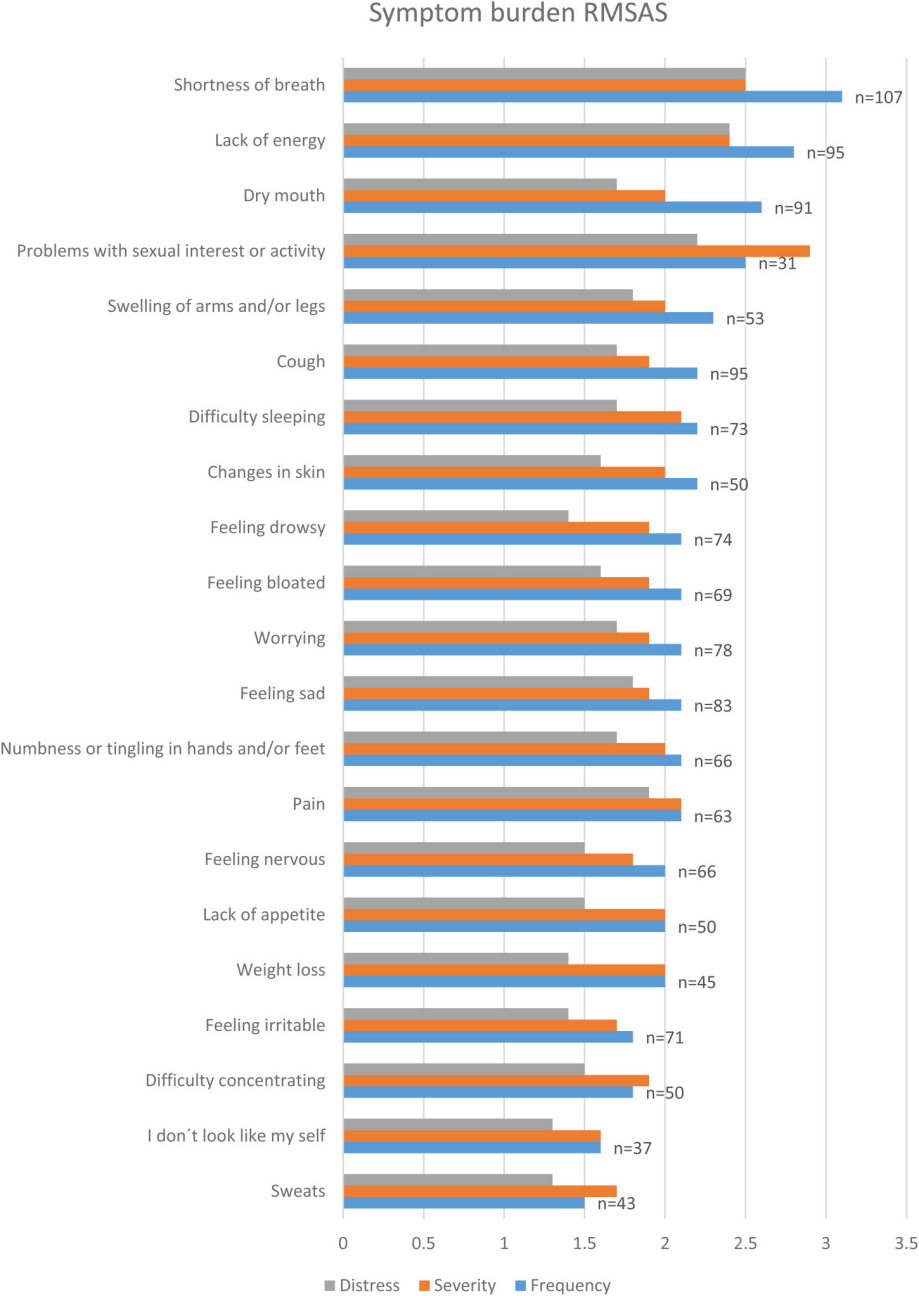
The symptom burden was estimated with Received Memorial Symptom Assessments Scale (RMSAS).

The three most prevalent symptoms scored with RMSAS based on frequency, severity, and distress were shortness of breath, lack of energy, and problems with sexual interest or activity. Problems with sexual interest or activity was scored high in severity, but there were only 31 persons who answered this question (28%). Dry mouth, swelling of arms and/or legs and cough were frequently reported symptoms. Pain and feeling sad were distressing symptoms (Figure 3).

The results are not shown but in data for the persons affected by COPD the symptoms vary from person to person and no significant differences can be seen between COPD stages III and IV. It seems that persons with COPD in stage III rate their symptoms as occurring more frequently than those in stage IV, but no significant difference can be seen except for the cough.

#### Caregiver burden

The caregiver burden reported by the next of kin has a total rating of 34–78 and a mean of 59.9. The caregiver burden is almost the same between men and women and stages of COPD (Table 2). On caregiver esteem men who are next of kin to persons in COPD stage III score higher with a range of 18–28. In both family support and finances the range is lower rated for all. This can indicate that there is a lack of support and an effect on the economy of being next of kin to a person with severe COPD. Low scores indicate high levels of caregiver burden.

**Table 2.** The Caregiver Reaction Assessment (CRA) from the next of kin.

	Stage III *n* Range Mean	Women	Men	Stage IV *n* Range Mean	Women	Men
Caregiver esteem 7 questions 7–28	**25**	**18**	**7**	**34**	**18**	**16**
	10–28	10–28	18–28	11–27	11–27	14–27
	22.3	21.8	23.6	22.4	21.8	23.0
Lack of family support 5 questions 5–20	**27**	**19**	**8**	**34**	**18**	**16**
	5–13	5–13	5–11	5–14	5–14	5–13
	7.9	8.1	7.5	7.9	8.2	7.4
Impact on finances 3 questions 3–12	**27**	**19**	**8**	**36**	**18**	**18**
	3–10	3–10	3–9	3–12	3–12	3–10
	4.7	4.7	4.8	5.3	5.6	5.1
Impact of schedule 5 questions 5–20	**30**	**21**	**9**	**35**	**18**	**17**
	5–15	5–13	6–15	5–18	5–18	5–13
	8.0	7.3	9.6	8.5	9.2	7.8
Impact on health 4 questions 4–16	**30**	**21**	**9**	**34**	**17**	**17**
	4–14	4–11	5–14	4–15	4–15	4–14
	6.9	6.6	7.4	6.7	7.1	6.3
Total 24 questions 24–96	**24**	**18**	**6**	**33**	**17**	**16**
	34–61	34–61	45–58	39–78	40–78	39–67
	49.3	49.0	50.0	50.4	51.6	49.2

Underline and bold figures are presenting the total number of next of kin assessing the CRA in the respective group.

#### Social support

For each question persons with COPD in stages III and IV and next of kin have almost the same number of persons around for support and almost the same level of satisfaction. Both affected persons and next of kin have a range of supportive persons, 0–9, but the mean is around two, which indicates that some are alone without supportive persons or only have one or two persons around for support (Table 3). For the affected persons (*n* = 85), 26 (31%) have a mean of no one or one person for support, and 27 (32%) affected persons have one or two persons. For the next of kin (*n* = 57) 16 (28%) have a mean of no one or one person for support and 20 (35%) next of kin have one or two persons.

**Table 3.** The SSQ-6 from persons affected by COPD and their next of kin.

	Item		1	2	3	4	5	6	Total item score	Total satisfaction score
Person with COPD	Number of persons	*n*	101	99	98	102	96	92	74	
		min-max	0–9	0–7	0–7	0–9	0–8	0–5	0–6	
		mean	2	2	2	2	2	2	2	
	Satisfaction	*n*	101	91	90	100	91	87		74
		min-max	1–6	1–6	2–6	2–6	1–6	1–6		2–6
		mean	5.4	5.4	5.4	5.5	5.4	5.3		5.5
Next of kin	Number of persons	*n*	64	63	63	65	63	63	58	
		min-max	0–9	0–5	0–7	0–9	0–9	0–9	0–7	
		mean	2	2	2	3	2	2	2	
	Satisfaction	*n*	60	57	61	62	60	60		52
		min-max	1–6	1–6	1–6	1–6	1–6	1–6		3–6
		mean	5.2	5.2	5.5	5.5	5.4	5.4		5.4

COPD: chronic obstructive pulmonary disease; SSQ-6: Social Support Questionnaire 6

#### Sense of coherence

The result is not shown, but in the data, persons in stage IV have slightly higher SOC than stage III, although there is no significant difference.

The next of kin have slightly higher SOC than those affected by COPD but there is no significant difference. Next of kin have a higher meaningfulness than persons affected by COPD but there is no significant difference. In total, the mean indicates a medium SOC of 62–79 (Table 4). For the affected person (*n* = 86), with a range of 22–77, 62 persons (72%) have low SOC, and 24 persons have medium SOC. The next of kin (*n* = 57), with a range of 44–79, 38 persons (67%) have a low SOC, and 19 persons have medium SOC. A low SOC is 61 and below, medium 62–79 and high 80–91.^24^

**Table 4.** The SOC of persons affected by COPD and their next of kin.

	Person with COPD *n * min-max mean	Next of kin *n * min-max mean
Meaningfulness	94	62
	7–28	10–28
	18.7	21.7
Comprehensibility	96	63
	12–35	11–35
	24.4	24.8
Manageability	98	65
	8–28	9–28
	20.8	20.0
Total	86	58
	35–91	34–88
	64.0	66.5

COPD: chronic obstructive pulmonary disease; SOC: sense of coherence.

### Findings from the interviews with the affected person and the next of kin

The findings from the interviews with the person affected by COPD show one theme, an altered everyday life^3^ and from the next of kin three themes, changed roles in daily life, putting life on hold and to stand aside.^7^ When comparing similarities and differences in the qualitative interview data^3,7^ it was recognized how intertwined the data on affected persons and next of kin was and that these impacted each other; The affected person must take the day as it comes, which also the next of kin confirm as their social activities being limited as they are not able to plan ahead. Next of kin need to be there assisting and supporting the affected person and taking on responsibilities. Support also includes giving the affected person time to do some activities, but at their own pace. The affected person must manage the symptoms and symptom burden, especially the breathing problems. This situation affects the next of kin, who give assistance with different treatments, support activities, and different aids. Standing aside and experiencing the stressful situation, when a significant other has breathing problems, makes them feel helpless. They experience caregiver burden, and they become physically and emotionally affected.

### Results from the mixed method

The quantitative and qualitative results presented have now been diverged or converged to aim for a broader and deeper understanding of how COPD affects symptom burden, caregiver burden the need for support, and sense of coherence for those involved. A visualized overview of this result is presented in Table 5.

**Table 5.** Integrated visual display of examples from the mixed method analysis.

Quantitative Results	Qualitative results	Mixed method results
Instrument and variable		
RMSAS Variables with the highest prevalence Shortness of breath, lack of energy, problems with sexual interest or activity CRA Lower-rated family support A caregiver burden in form of lack of support.	An altered everyday life for the affected by COPD The next of kin must put the life on hold Standing aside	The symptom burden affects the person affected by COPD and the next of kin with limitations in daily life and social interaction Both the affected persons and the next of kin take the day as it comes and have to deal with the symptoms Next of kin is physically and emotionally affected Next of kin will be there and help and at the same time have time for their own life
SSQ-6 Affected persons have a supportive person around but are not satisfied The next of kin have one or two persons around and are satisfied	Need of support A well-balanced support To be there and support	Persons affected by COPD have few persons who support them and low support but are satisfied, next of kin have support but low satisfaction A balance between the need of support for the affected person and the possibility to give support for the next of kin and the balance with life. The affected person needs support but wants to do some activities by themself when it is possible. The next of kin want to help and take sometimes over more than needed
SOC 62 of the affected persons have low SOC and 24 have medium SOC 38 of the next of kin have a low SOC and 19 medium SOC		Both persons affected by COPD and next of kin have a low SOC which affects their whole life situation together with the different perspectives of burden.

COPD: chronic obstructive pulmonary disease; RMSAS: Received Memorial Symptom Assessments Scale; CRA: Caregiver Reaction Assessment; SSQ-6: Social Support Questionnaire 6; SOC: sense of coherence

## Discussion and practice implications

In this study, we found in the quantitative analyses that the symptom burden estimated with RMSAS showed that all the symptoms were different and tended to vary irrespective of the stage of COPD. This is supported by the fact that the affected persons say that they must take the day as it comes because of the symptom of breathing problems and its consequences. Even the next of kin assesses the caregiver burden in the form of caregiver esteem high: They are affected by the symptoms of the affected person and experience that they also must take the day as it comes. All this is in line with the concept of caregiver burden defined as self-perception, multifaceted strain, and as continuing over time.^5^ For both the affected and next of kin we can see a need to plan their daily living, including social activities. Affected person and next of kin affecting each other and adding effects to the caregiver burden which leads to issues the next of kin must deal with.^6^ Next of kin to persons with COPD in this study showed that seeing the symptom burden and the progression of the disease and experiencing feelings of not being able to do anything entails a caregiver burden. These emotions could worsen as the SOC was rated low both for affected persons and next of kin. SOC is important for how the affected person with COPD and the next of kin use the disease experience and knowledge in order to manage their life situation.^7,25^ Adding to these facts, our results showed that both affected and next of kin have few people around to get support from. Despite the low number of supportive persons, both affected persons and next of kin are satisfied with the situation. This situation could only be speculated about; it may be an effect of the chronic irreversible COPD diagnosis worsening over time and the four stages of COPD leading to adaptation to living with the disease. This shows the complexity and the effects of the disease, which GOLD^1^ also describes.

In this study, the next of kin rate a caregiver burden and experience an affected life situation, with limitations in social interactions and activities. They experience that the affected person needs help, and this help should be provided by the next of kin. The next of kin will be there to take care of the affected persons and sometimes the next of kin take over and become protective from the affected person's perspective. The affected person who is experiencing the symptom burden does not want to be another burden to the next of kin. The affected persons said that this support needs to be well balanced. A majority of both affected persons and next of kin have a low SOC and the next of kin said that they needed more information about the disease and its progression of it to make it understandable and manageable. Regarding facilitation, the next of kin report that they have persons who support them, but they are not as satisfied with this as the affected persons. From this, we can see that the affected person has support even if it is from few persons. Support for the next of kin needs development.

In this mixed method results, we can see various dimensions of the same situation and several perspectives, both from estimations and experiences but also from the perspective of the diagnosed person and the perspective of the next of kin. Lan et al.^26^ also see a need for support from the next of kin but in the form of help in using available resources to improve the level of self-management. From this, we can see that the symptom and caregiver burden affect the next of kin continuously and over a long-time frame. Looking at separate results gives one point of view, but together with other instruments and qualitative data, there is another life situation shown a more complex life situation. This leads to a need for person-centered interventions with information, knowledge, support and understanding adapted for this specific situation, which helps to develop the capacity to manage this complex situation. All this is in line with how understandable the situation is, whether there is meaningfulness and whether the situation is manageable, then there is a SOC.^8^ From this we can see that person-centered care can facilitate self-care through knowledge of self-care activities. The goal of self-care is to prepare optimal functions through knowledge, skills training and motivated engagement.^27^

This study has both strengths and limitations. The strength of this study lies in the inclusion of both the affected person's and next of kin's perspectives when considering experiences of the effects of COPD. Using a mixed method to divergent and convergent results (confirm or disconfirm), broaden the perspectives. This integration and interpretation lead to a strengthened rigour. Limitations are that fewer next of kin than persons with COPD answered the questionnaires and that no reminder could be sent out.

## Conclusions

In summary, we found that persons affected by COPD and their next of kin have a symptom and caregiver burden affecting their life situation. To manage this situation there is a need for increased support. With few persons around to give support and with a low SOC, regarding comprehensibility and meaningfulness it is hard to manage the situation. Together this leads to a need for improved person-centered interventions.
